# Clinical Profiling of BCL-2 Family Members in the Setting of BRAF Inhibition Offers a Rationale for Targeting *De Novo* Resistance Using BH3 Mimetics

**DOI:** 10.1371/journal.pone.0101286

**Published:** 2014-07-01

**Authors:** Dennie T. Frederick, Roberto A. Salas Fragomeni, Aislyn Schalck, Isabel Ferreiro-Neira, Taylor Hoff, Zachary A. Cooper, Rizwan Haq, David J. Panka, Lawrence N. Kwong, Michael A. Davies, James C. Cusack, Keith T. Flaherty, David E. Fisher, James W. Mier, Jennifer A. Wargo, Ryan J. Sullivan

**Affiliations:** 1 Division of Surgical Oncology, Massachusetts General Hospital, Boston, Massachusetts, United States of America; 2 Harvard Medical School, Boston, Massachusetts, United States of America; 3 Division of Medical Oncology, Massachusetts General Hospital Cancer Center, Boston, Massachusetts, United States of America; 4 Department of Surgical Oncology and Genomic Medicine, University of Texas, M.D.Anderson Cancer Center, Houston, Texas, United States of America; 5 Division of Hematology Oncology, Beth Israel Deaconess Medical Center, Boston, Massachusetts, United States of America; 6 Department of Dermatology, Massachusetts General Hospital, Boston, Massachusetts, United States of America; The Moffitt Cancer Center & Research Institute, United States of America

## Abstract

While response rates to BRAF inhibitiors (BRAFi) are high, disease progression emerges quickly. One strategy to delay the onset of resistance is to target anti-apoptotic proteins such as BCL-2, known to be associated with a poor prognosis. We analyzed BCL-2 family member expression levels of 34 samples from 17 patients collected before and 10 to 14 days after treatment initiation with either vemurafenib or dabrafenib/trametinib combination. The observed changes in mRNA and protein levels with BRAFi treatment led us to hypothesize that combining BRAFi with a BCL-2 inhibitor (the BH3-mimetic navitoclax) would improve outcome. We tested this hypothesis in cell lines and in mice. Pretreatment mRNA levels of *BCL-2* negatively correlated with maximal tumor regression. Early increases in mRNA levels were seen in *BIM*, *BCL-XL*, *BID* and *BCL2-W*, as were decreases in *MCL-1* and *BCL2A*. No significant changes were observed with *BCL-2*. Using reverse phase protein array (RPPA), significant increases in protein levels were found in BIM and BID. No changes in mRNA or protein correlated with response. Concurrent BRAF (PLX4720) and BCL2 (navitoclax) inhibition synergistically reduced viability in BRAF mutant cell lines and correlated with down-modulation of MCL-1 and BIM induction after PLX4720 treatment. In xenograft models, navitoclax enhanced the efficacy of PLX4720. The combination of a selective BRAF inhibitor with a BH3-mimetic promises to be an important therapeutic strategy capable of enhancing the clinical efficacy of BRAF inhibition in many patients that might otherwise succumb quickly to *de novo* resistance.

**Trial Registrations**: ClinicalTrials.gov NCT01006980;

ClinicalTrials.gov NCT01107418;

ClinicalTrials.gov NCT01264380;

ClinicalTrials.gov NCT01248936;

ClinicalTrials.gov NCT00949702;

ClinicalTrials.gov NCT01072175

## Introduction

Vemurafenib and dabrafenib are selective BRAF inhibitors that improve overall survival when compared with dacarbazine in patients with advanced, BRAF-mutant melanoma [1], [2]. While these results have changed the standard of care for these patients, there remain critical limitations to the activity of these agents. Specifically, clinical resistance develops in most patients within one year, the median progression free survival (PFS) is 5–6 months, and durable remissions are uncommon [1]–[5]. Acquired resistance to BRAFi therapy is mediated by multiple mechanisms that lead to reactivation of the mitogen activated protein kinase (MAPK) pathway or upregulation of other pro-survival signaling pathways [e.g. phosphoinositide-3-kinase (PI3K) pathway] [6]–[17] While less is known about *de novo* resistance to therapy, stromal production of HGF and PTEN deficiency each have been shown to be associated with poorer outcomes through unopposed PI3K pathway activity. Another recently described mechanism of *de novo* resistance to BRAFi therapy is dysregulation of the cell cycle, either through overexpression of *CCND1* (cyclin D1) or loss of the cyclin dependent kinase inhibitor, *CCDNK2A* (p16^INK4A^). Finally, our group has recently described that high BCL2A1 (an anti-apoptotic BCL-2 family member) expression is associated with resistance to BRAFi-induced apoptosis *in vitro* and with a lower response rate in patients treated with a BRAFi [17], [18].

BCL-2 family proteins are major regulators of the apoptotic threshold and are deregulated in many cancer types [19]. The anti-apoptotic members of the BCL-2 family, known as multi-domain anti-apoptotic proteins, include: BCL-2, BCL2-L1 (BCL-XL), BCL2-L2 (BCL-W), MCL-1, and BCL-2A1 (BFL-1). In melanoma, altered BCL-2, BCL-XL, and MCL-1 expression are associated with malignant transformation of melanocytic cells and progression to melanoma [20]. In addition, increased expression of BCL-XL is associated with a poor prognosis in patients with melanoma and elevated BCL-2 and BCL-XL are associated with a poor response to chemotherapy [21]–[23].

Over-expression of the multi-domain anti-apoptotic proteins contributes to apoptosis resistance in multiple types of cancer including melanoma. However, there are a number of pro-apoptotic BCL-2 family members that facilitate apoptosis through inhibiting the anti-apoptotic family members and activating the mitochondrial cell death pathway. The two multi-domain pro-apoptotic proteins, BAK and BAX, reside in the outer mitochondrial membrane and, when activated, lead to the depolarization of the mitochondria and the subsequent release of cytochrome C, as well as other mediators of apoptosis. Activation of BAK and BAX is mediated through interactions with a third class of BCL-2 family members known as the BCL-2 Homology 3 domain (BH3) only proteins. The activator BH3-only proteins, BID and BIM, initiate apoptosis by binding directly to BAK and BAX. Other BH3-only proteins, however, such as BAD, BMF, BIK, HRK, NOXA and PUMA, are able to bind and regulate (or be regulated by) the anti-apoptotic BCL-2 proteins [24].

One potential way to enhance the effectiveness of BRAF-directed therapy is to focus on mechanisms that lower the threshold for apoptotic induction by MAPK pathway inhibitors. Mutant BRAF modulates proapoptotic BCL-2 family members, including the inactivation of BAD and downregulation of BIM, serving to protect the cell from apoptosis [25], [26]. In preclinical models, inhibition of BRAF or MEK, either through small interfering RNA (siRNA) or small molecule inhibitors, initiates both growth arrest and apoptosis. This is at least in part caused by upregulation of BIM and its associated suppression of two anti-apoptotic BCL-2 family members, BCL-2 and MCL-1 [27], [28]. In patients, single agent BRAFi therapy is associated with inconsistent induction of apoptosis that is not associated with clinical outcome [29], [30]. We hypothesized that BRAF inhibitor therapy would modulate both pro- and anti-apoptotic BCL-2 family members and sought to investigate the effects of BRAF-directed therapy on the RNA and protein expression of BCL-2 family members, by comparing pre- and on-treatment biopsies of patients with BRAF mutant melanoma treated with either single-agent vemurafenib or the combination of dabrafenib and trametinib. Furthermore, we evaluated the cytotoxic effects of combining a BH3-mimetic, navitoclax, with a BRAF inhibitor *in vitro* and *in vivo* in melanoma cell lines.

## Materials and Methods

### Patient samples

Patients with metastatic melanoma containing BRAF^V600E^ mutation (confirmed by genotyping) were enrolled on clinical trials for treatment with a BRAF inhibitor (vemurafenib) or combined BRAF + MEK inhibitor (dabrafenib + trametinib; Table S1). All samples were obtained from participants who signed an informed consent form. The current IRB approval letter has been attached. This protocol was reviewed and approved by the Dana-Farber Cancer Institute (DFCI) IRB, in accordance with the applicable Federal regulations set forth at 45 CFR Part 46, and 21 CFR Parts 50 and 56. All relevant clinical trials are registered at ClinicalTrials.gov. ClinicalTrials.gov numbers are as follows: NCT01006980, NCT01107418, NCT01264380, NCT01248936, NCT00949702, and NCT01072175. Tumor biopsies were conducted pretreatment (day 0) and at 10 to 14 days on treatment. Formalin-fixed tissue was analyzed to confirm that viable tumor was present via hematoxylin and eosin (H&E) staining. Additional tissue was snap-frozen and stored in liquid nitrogen or was immediately processed for purification of RNA.

### Clinical Response

RECIST criteria were used to classify response, and are defined as follows: Complete Response (CR): Disappearance of all target lesions. Partial Response (PR): At least a 30% decrease in the sum of the longest diameter (LD) of target lesions, taking as reference the baseline sum LD. Stable Disease (SD): Neither sufficient shrinkage to qualify for PR nor sufficient increase to qualify for PD, taking as reference the smallest sum LD since the treatment started. Progressive Disease (PD): At least a 20% increase in the sum of the LD of target lesions, taking as reference the smallest sum LD recorded since the treatment started or the appearance of one or more new lesions.

### Purification of total RNA

Patient samples were homogenized and disrupted using a mortar and pestle followed by use of a QIAshredder. A QIAcube was used to harvest RNA from both patient biopsies and cell lysates using the RNeasy Mini Protocol (Qiagen).

### Quantitative PCR

Total RNA (250 ng) was used as template, and Superscript VILO cDNA Synthesis Kit (Invitrogen) was used to generate cDNA. Quantitative real-time PCR was carried out on an Applied Biosystems 7300 machine. Primers for PCR are described in Table S2.

### RPPA

Protein lysates were isolated from SNAP frozen patient tumors and processed for reverse phase protein array (Methods RPPA S1).

### Cell lines and reagents

Malignant melanoma cell lines were obtained from the American Type Culture Collection (ATCC) and kindly donated by the Department of Dermatology (Hensin Tsao lab) from the Massachusetts General Hospital (MGH) between 2011 and 2012. Cells were authenticated following ATCC recommendations (ATCC Tech bulletin #8), and used within one week after authentication. Cells were passaged for less than six months after received. Cell morphology and growth analysis were performed posterior to resuscitation. All cell lines were maintained and all experiments were performed in DMEM (Sigma, D 6429) supplemented with 10% (v/v) FBS (Gibco) and 1% (v/v) penicillin-streptomycin (Life Technologies). For *in vitro* studies, BRAF inhibitor, PLX4720 (Selleck Chem), and BCL-2/BCL-XL inhibitor, ABT-263 (Selleck Chem) were used.

### Cell Proliferation Assays

Cellular proliferation was evaluated by MTT assay (3-(4,5-dimethylthiazole- 2-yl)-2,5-diphenyl- 2H-tetrazolium bromide; Sigma-Aldrich) following manufacturer instructions. Cells were plated in 96-well plates at 1,000 to 10,000 cells per well in 100 µL of media and treated 24 hours after plating. MTT signal was read at 72 hours after treatment. The IC_50_ and Combination Index (CI) by Chou-Talalay were determined from the regression plot logarithm of the concentration versus effect using Calcusyn Software (Biosoft) v1.1. In addition, conservative isobolograms were used to show synergism and/or antagonism.

### Immunoblotting

Immunoblotting was performed using Cell Signaling general protocols, antibodies: Cell Signaling; MCL1 Rabbit Ab 4572S, BIM (C34C5) Rabbit Ab, #2933S, GAPDH (14C10) Rabbit Ab #2118, Millipore; β-Tubulin (KMX-1) Mouse Ab #3408.

### Annexin:PI apoptosis assay by flow cytometry

Annexin V staining was performed using the Annexin-V-FITC apoptosis detection kit (BD Pharmingen) following manufacturer's protocol in package insert. 5×10^5^ cells were seeded into 6 well plates in DMEM and 10% FBS (GIBCO) and Pen-strep (Life Technologies) 24 hours prior to treatment. Cells were treated for 24 hours with the specified drug concentration. After harvesting by trypsinization, cells were washed twice with cold phosphate-buffered saline. 3×10^4^ cells were resuspended in 100 µl of 1X Binding Buffer and stained with 5 µl of propidium iodide (PI) and 5 µl of annexin-V solution for 15 min at RT in the dark. After incubation, 400 µl of 1X Binding Buffer was added to each tube and the samples were examined by flow cytometry (FACS calibur). Controls (unstained cells and single-stained cells with Annexin V or PI) enabled the compensation and definition of quadrants for posterior analysis using WinMDI 2.9.

### Xenograft Model

Athymic nude mice Nu/Nu (Crl:NU-Foxn1nu), 4 weeks of age were purchased from Charles River Laboratories and Taconic Farms. All animal work was conducted according to relevant national and international guidelines under a Beth Israel Deaconess Medical Center Animal Care and Use Committee approved document. Cell lines A375 and A2058 (BRAF V600E mutant) were obtained from the American Type Culture Collection (ATCC) and authenticated by isoenzymology. The Cytochrome C subunit I (COI) PCR assay was performed for confirmation of species and cell line had identity was confirmed by STR analyses. Cells were injected SC at 5×10^6^ cells per mouse. Mice were randomized once an average of ∼300 mm^3^ tumor volume was reached (For A375 and A2058, average time was 7 to 9 days). After randomization, treatment was started. Mice were sacrificed following the guidelines by the Institutional Animal Care and Use Committee (IACUC) for MGH. Tumor volumes were determined using [D× (d^2^)]/2, in which D represents the largest diameter of the tumor, and d represents the largest perpendicular volume to D. Tumor volumes were normalized individually to their initial volume (Volume at treatment day 1) (Relative tumor Volume  = V_x_/V_0_; were V_x_ corresponds to the volume for the specific animal at a particular day and V_0_ corresponds to the initial volume for the given animal. BRAF inhibitor, PLX4720 (Selleck), was diluted in DMSO to a 20 mg/mL stock. Stock was diluted in 1% Methyl-cellulose (10 mg Methyl-cellulose/mL of H2O) for daily oral gavage at 100 mg/kg/day. Navitoclax (ABT-263) was dissolved in 60% *Phosal 50 PG (American Lecithin)*, 30% polyethylene glycol 400, 10% EtOH vehicle and administered PO at 100 mg/kg/day. Statistical analysis consisted of Mann-Whitney Rank Sum Test, two-way ANOVA, and post hoc Bonferroni using GraphPad Prism, version 4.3.

### IHC in Xenografts

Tumors were fixed in 10% neutral buffered formalin, embedded in paraffin, and sectioned at five microns 3 days after treatment commenced. Deparaffinized and rehydrated sections were subjected to epitope retrieval in 10 mM Tris-EDTA buffer pH 9.0 and blocking in 3% BSA in TBST (Tris pH 7.6, 0.05% Tween-20). BIM (Cell Signaling C34C5) Rabbit Ab (1∶100 in 3% BSA in TBST) was applied for 1 hr at RT. After peroxidase block in 3% H_2_O_2_, HRP- labeled anti-rabbit secondary antibody (Dako EnVision, K4003, RTU) was applied for 30 minutes. Slides were developed with DAB+ (Dako K3468) and counterstained with hematoxylin (Vector H-3401) prior to dehydration and mounting.

### Apoptosis in Xenografts

Tumors were fixed in 10% neutral buffered formalin, embedded in paraffin, and sectioned at five microns 3 days after treatment commenced. Deparaffinized and rehydrated sections were subsequently processed using the TACS TdT DAB Kit protocol (Catalog #4810-30-K) and visualized by light microscopy.

### Statistical Analysis

If not indicated otherwise, data represent results for assays performed in triplicate, with error bars to represent standard errors from the mean. All box plots and linear regressions performed using the R statistical package.

## Results

### *BCL-2* expression correlates with tumor response after BRAF inhibition in melanoma

**Figure 1:**
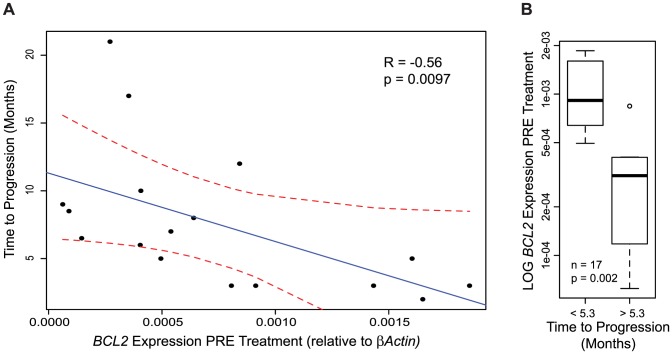
Pre treatment *BCL-2* expression in tumors of patients with metastatic melanoma negatively correlates with response to BRAF inhibition. (**A**) Linear regression between duration of patient response to BRAF inhibition and *BCL-2* expression levels relative to endogenous gene *βActin* prior to treatment (n = 17, R = −0.56, p = 0.0097, dotted red lines indicate 95% confidence intervals). (**B**) Patients who progress in less than 5.3 months have higher levels of *BCL2* mRNA expression relative to *βActin* pre-treatment than patients with an above average duration of response to BRAF inhibition (P≤0.05).

We evaluated pretreatment mRNA expression of BCL-2 family members, *BCL-2*, *MCL-1*, *BCL-XL, BCL-W*, *BIM*, and *BID* in tumors of patients with metastatic melanoma. Pre-treatment expression levels of *BCL-2* in melanoma patient samples inversely correlated with tumor response after BRAF inhibition (Figures 1A and 1B), while pretreatment levels of the other assayed BCL-2 family members had no correlation with outcome (Figure S1).

### Change in mRNA and protein expression of BCL-2 family members and markers of apoptosis in pre- and post- BRAF inhibition in tumor samples

**Figure 2:**
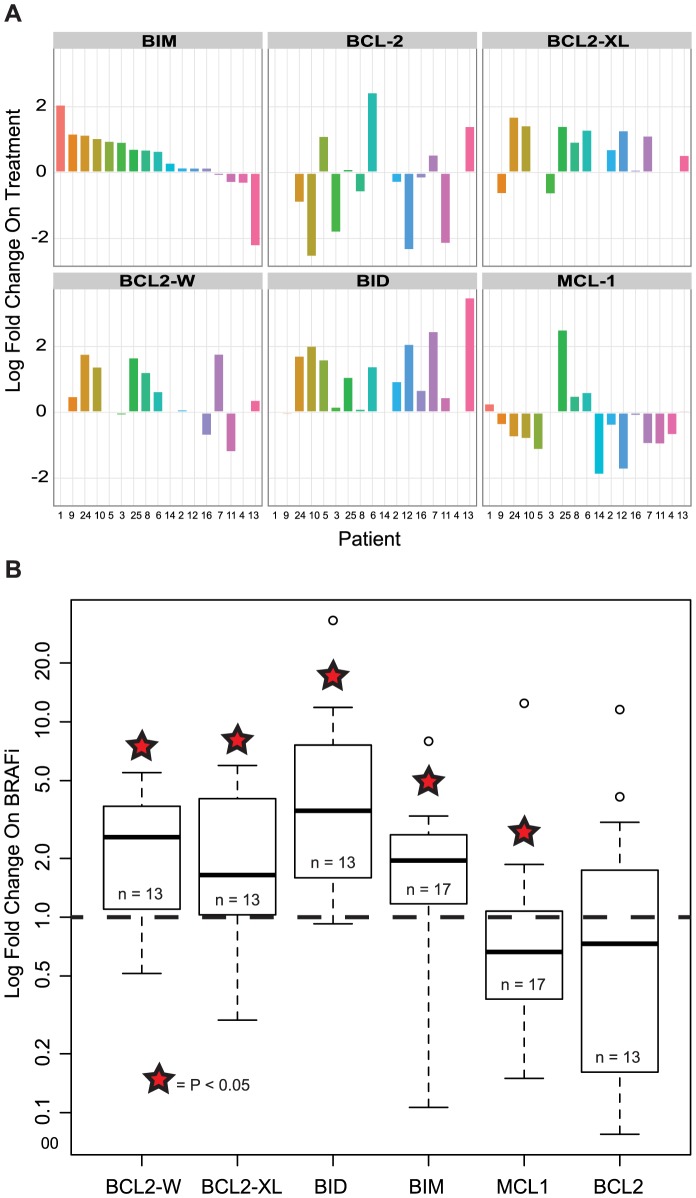
BRAF inhibition is associated with changes in BCL2 family member expression in tumors of patients with metastatic melanoma. mRNA levels of *BIM*, *BCL2-XL*, *BCL2-W*, *BID* increased in patients with metastatic melanoma undergoing treatment with a selective inhibitor of BRAF^V600E^ while mRNA levels of *MCL-1* decreased; *BCL2* levels did not change significantly across patients. (**A**) mRNA expression levels of each gene from pre and on treatment biopsies from each patient were quantified by real-time PCR and are plotted as log fold change. Each number along the x-axis indicates an individual patient identifier, the y-axis indicates the mRNA level changes of BCL-2 family members for each patient. (**B**) Changes in mRNA expression levels across patients 10–14 days after initiation of BRAFi are plotted on a log scale as fold change from pre-treatment levels using box and whisker plots (* = P≤0.05).

**Figure 3:**
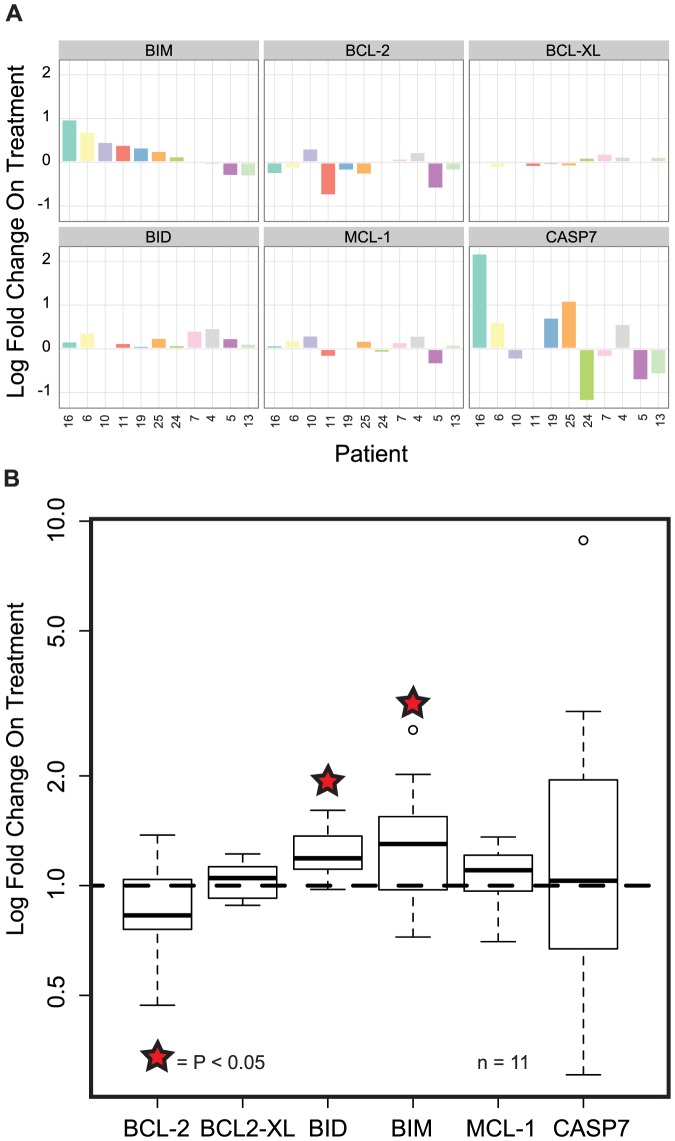
Protein expression levels of BCL2 family members in patients undergoing treatment with a BRAF inhibitor. RPPA analysis of tumors from patients with metastatic melanoma shows a significant increase of BID and BIM on BRAFi. (**A**) Protein expression levels of each gene from pre and on treatment biopsies for each patient are shown as log fold change on treatment. (**B**) Changes in protein expression levels across patients 10–14 days after initiation of BRAFi are plotted on a log scale as fold change from pre-treatment levels using box and whisker plots (* = P≤0.05).

We tested the mRNA and protein expression of *BCL-2* family members in tumor samples from patients with metastatic melanoma 10–14 days into treatment with a BRAF inhibitor. We found no significant change in *BCL-2* mRNA expression upon treatment. However, we found a statistically significant increase in expression of *BIM*, *BCL-XL*, *BCL-W*, and *BID* mRNA in samples from patients upon BRAF inhibition as well as a significant reduction in *MCL-1* (Figures 2A and 2B) [31]. There was no correlation between clinical benefit and the magnitude of change in mRNA levels of any of these genes after BRAF inhibition. Protein expression of the BCL-2 family members MCL-1, BCL-2, BCL-XL and BCL-W were not significantly changed while BIM and BID levels increased in the setting of BRAFi therapy (Figures 3A and 3B). Similarly, there is no significant increase of CASP7 in the setting of BRAFi therapy, though there is significant variability of effects on CASP7 in individual patient samples.

### *In vitro* mutant BRAF inhibition modulates BIM and MCL-1 expression similarly to clinical BRAF inhibition in patients with BRAF-mutant melanoma

**Figure 4:**
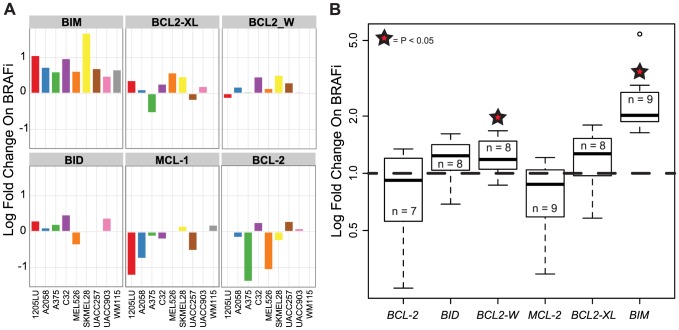
Expression levels of BCL2 family members in a panel of BRAF mutant cell lines undergoing BRAF inhibition. (**A**) mRNA expression levels of various BCL2 family members were quantified by real-time PCR changes and are plotted as log fold difference from vector control (DMSO). (**B**) Across our panel of cell lines, *BCL2-W* and *BIM* increased significantly from control in the context of BRAF inhibition. PLX4720 (1 µM) was used as BRAF inhibitor.

**Figure 5:**
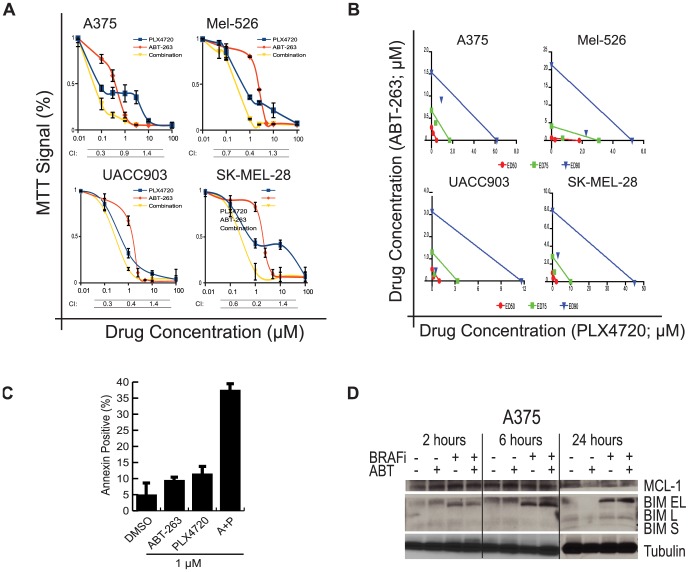
The effect of BRAF inhibition, BH3-mimetics or their combination on cell proliferation, apoptosis and protein expression levels of BCL2 family members in BRAF^V600E^ melanoma cell lines. (**A**) MTT assay demonstrating the effect of BCL-2 inhibition, BRAF inhibition, and their combination, on cell proliferation with their respective combination index (CI) value. (**B**) Corresponding isobolograms. (**C**) Fluorescence activated cell-sorting (FACS) for Annexin after indicated drug treatment in a BRAF^V600^ cell line, A375. Drug combinations used at a 1∶1 ratio. (**D**) Western blotting of BIM and MCL1 in a BRAF^V600^ cell line, A375 after 2, 6 and 24 hours treatment with a BRAFi, ABT and the combination of both BRAFi and ABT.

We assayed a panel of BRAF^V600E^ mutant human melanoma cell lines for the same group of BCL2 family members and found them to recapitulate the responses found in patients. BRAF inhibition of the MAPK pathway resulted in significant increases in mRNA levels of *BIM* and *BCL2-W* (p<0.05) (Figures 4A and 4B), consistent with clinical samples. Increased levels of *BID* and *BCL2-*XL (p<0.08) and decreased mRNA levels of *MCL-1* (p<0.08) were also observed *in vitro* after treatment with BRAF inhibition in most but not all cell lines. Also consistent with patient samples, *BCL2* levels did not change across cell lines (Figures 4A and 4B). Using the Broad-Novartis Cancer Cell Line Encyclopedia we did not see a correlation, however, between BCL2 levels pre-treatment and sensitivity to BRAF inhibition as measured by IC50 across 22 BRAF mutant melanoma cell lines. Protein levels of BIM and MCL-1 were probed across our panel of cell lines (Figure 5D and Figure S2B) at 2, 6 and 24 hours after treatment and were found to be consistent with the RNA results.

### Concomitant inhibition of both BCL-2 and mutant BRAF synergistically suppress cell growth and augment apoptosis

Having observed that higher pre-treatment levels of BCL2 were a predictor of poor response to BRAFi and that the anti-apoptotic BCL2-XL increases in the context of BRAFi, we predicted that combining BRAF inhibition with BCL2/BCL2-XL inhibition would mitigate resistance to BRAFi. We evaluated cell growth in melanoma cell lines after BCL-2/BCL-XL inhibition with ABT-263 and BRAF inhibition with PLX4720 using MTT assays. Both inhibitors reduced cell viability in a dose related manner (Figures 5A and B). The effect of the combination was greatest between 48 and 72 hours of treatment. Using Chou-Talaly method for combination studies, we found that the combination of both inhibitors results in a synergistic reduction of cell growth in the tested cell lines in a broad range of combination ratios (i.e. 1∶1; 1∶2 and 1∶10) as evidenced by combination index studies (Figures 5A and 5B) [32]. We directly assessed apoptosis following BH3 mimetic and BRAF treatment therapy using Annexin:PI by fluorescence activated cell sorting (FACS). As reported by others, the effects of BRAF inhibition on apoptosis is dose- and cell line dependent [33], [34]. As expected, despite increased levels of BIM, the resulting increase in apoptosis seems to be modest (Figure 5C). As monotherapy, the effects of ABT-263 on apoptosis were comparable to that observed with BRAF inhibition, suggesting the need for an additional stimuli to induce apoptosis. The combination of both inhibitors results in an increase of apoptosis and cell death in a dose- and cell line dependent manner (Figure 5C and Figure S2A).

### BH-3 mimetic treatment, BRAF inhibition, and their combination reduce tumor growth in BRAF V600E mutant xenografts

**Figure 6:**
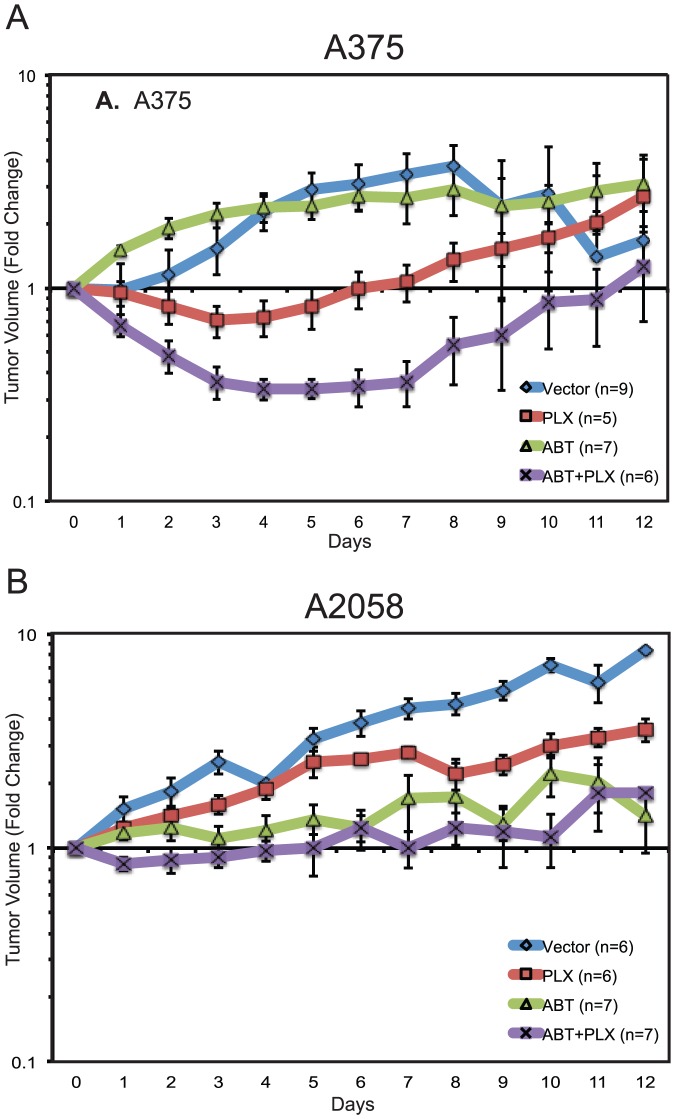
The effect of BH-3 mimetic treatment, BRAF inhibition, and their combination on tumor growth in BRAF V600E mutant xenografts. (**A**) A375 xenograft. (**B**) A2058 xenograft. PLX4720 was used as BRAF inhibitor and ABT-263 was used as BH3-mimetic. Both inhibitors were given to mice PO daily at 100 mg/kg [45] for 12 days according to treatment group. Mice were euthanized when tumors reached maximal allowed tumor volume. This occurred between days 9 and 12 for some but not all animals. Error bars represent standard error of the mean (SEM).

We evaluated the effects of navitoclax and PLX4720 or their combination *in vivo*. Oral administration of compounds ABT-263 or PLX4720 were used to produce BCL-2 and BRAF inhibition in mice. We used 100 mg/kg/d PO daily as our treatment dose and schedule. As single therapy, both ABT-263 and PLX4720 suppressed tumor growth in A375 and A2058 xenograft mouse models (Figures 6A and 6B). Both inhibitors were well tolerated with no overt toxic effects or weight loss. The combination of ABT-263 and PLX4720 was well tolerated and resulted in enhanced tumor growth suppression in both sensitive and resistance models. BIM levels were increased in these tumors and TUNEL staining confirmed the presence of at least some cells undergoing apoptosis (Figure S3). Greater tumor regression was observed for the combination in the sensitive model, however no complete responses were observed.

## Discussion

Our analysis of serial tumor biopsies in patients with BRAF-mutant melanoma treated with BRAFi has revealed a number of findings. First, in a panel of four anti-apoptotic BCL-2 family members, only pretreatment levels of BCL-2 are associated with outcome to BRAFi treatment; in particular, elevated BCL-2 levels significantly correlated with a poorer outcome. Second, BRAFi treatment results in significant increases in mRNA and protein levels of the proapoptotic BH3-only proteins BIM and BID. Third, BRAFi therapy drives a decrease in mRNA levels of MCL-1, but an increase in the mRNA levels of BCL-XL and BCL-W; though it is important to note that these mRNA changes did not translate into significant changes in the level of protein expression of each of these anti-apoptotic BCL-2 family members when using the RPPA platform. Fourth, with the exception of pretreatment BCL-2 levels, neither the pretreatment mRNA and protein levels of both pro- and anti-apoptotic BCL-2 family members nor the magnitude of change in these mRNA levels are associated with clinical outcome. Finally, *in vitro* changes in mRNA expression of many anti- and pro-apoptotic family members from several BRAF-mutant melanoma cell lines mirror the changes seen in samples obtained from patients treated with BRAFi; thereby validating the use of these cell lines to evaluate the efficacy of combinatorial strategies targeting apoptosis.

These findings support the evaluation of the BH3-mimetic navitoclax (ABT-263) and its tool compound, ABT-737, in combination with BRAF inhibition as a strategy to improve the effectiveness of BRAF inhibitors; as the targets of these agents, the anti-apoptotic proteins BCL-2, BCL-XL, and BCL-W, either are associated with outcome (BCL-2) to or increased in response (BCL-XL and BCL-W) to BRAF inhibition [35], [36]. Importantly, the cell lines most resistant to the BH3-mimetics show overexpression of MCL-1, and pharmacologic downregulation of MCL-1 potentiates the lethality of ABT-737 in these cell lines [37], [38]. As MCL-1 expression is reduced and its binding partners BID and BIM are increased with BRAFi treatment, it is predicted that MCL-1 would be sufficiently abrogated by the changes of BRAF inhibition. Providing further support of this strategy, preclinical studies of ABT-737 in combination with a MEK inhibitor led to enhanced lethality, *in vitro* and *in vivo* utilizing BRAF-mutant melanoma cell lines and xenografts respectively, compared to either agent alone [39]. Additionally, ABT-737 in combination with the BRAF inhibitor PLX4720 showed augmentation of apoptosis in BRAF mutant melanoma cell lines that was BIM dependent and mediated through an enhancement of the BIM:MCL-1 interaction [40]. We provide confirmation *in vitro* that combining navitoclax with a BRAF inhibitor is clearly synergistic with respect to inhibiting cell growth, and is associated with a dramatic increase in apoptosis. Further, we show *in vivo* that the combination is associated with deeper and more prolonged regression compared with the single-agent BRAF inhibition and single-agent navitoclax in both a BRAF inhibitor sensitive cell line (A375) and a BRAF inhibitor resistant cell line (A2058).

Our clinical samples were obtained from patients who received either single-agent vemurafenib or the combination of dabrafenib and trametinib, yet are pooled together for this analysis. While there is little difference in either the inhibition of the MAPK pathway (pERK) or the changes in mediators of apoptosis in these distinct patient subgroups at the biopsy time-point (10–14 days), it is very possible that differences in single-agent BRAFi or combination BRAFi and MEKi at that time-point may exist and influence and therefore limit these findings. Additionally, we have chosen not to focus on BCL2A1, a recently described melanoma oncogene and mediator of BRAF inhibitor resistance in a subset of melanomas, as this was the subject of an independent analysis in our program [34]. This work cautions that a subset of patients likely will not benefit from the combination of navitoclax with a BRAF inhibitor regimen, though also opens the possibility that BCL2A1 expression or change in expression may be a useful biomarker to study in clinical trials of navitoclax in melanoma and may become a biomarker used to exclude patients from future clinical trials with navitoclax in melanoma. It should be noted that obatoclax, another BH3-mimetic that targets BCL-2, BCL-XL, and BCL-W but also MCL-1 and BCL2A1, has been shown to enhance the activity of BRAF inhibitors in preclinical studies, however human studies with obatoclax have been limited by the CNS toxicity profile of this agent and there are currently no open clinical trials studying obatoclax according to clinicaltrials.gov [41]–[44].

For the first time, we demonstrate that BCL-2 expression is inversely associated with patient outcome in the context of targeted BRAFi therapy. Furthermore, we detail the changes in expression of several key regulators of apoptosis in BRAF-mutant melanoma in the setting of BRAF inhibition. We confirm the *in vitro* synergy of BRAF inhibition with a BH-3 mimetic and demonstrate improved outcome for this combination *in vivo*. All these findings support the development of clinical trials evaluating the role of adding agents that target apoptosis, generally, and BH3-mimetics, specifically, in combination with BRAF-directed therapy in patients with BRAF-mutant melanoma. The first of these trials has recently opened (NCT01989585) and is a Cancer Therapeutics Evaluation Program (CTEP)-sponsored study of navitoclax in combination with the BRAF inhibitor dabrafenib and the MEK inhibitor trametinib in BRAF-mutant melanoma. Our data predict that we will see variability of response to this combination, but that we will likely see an enhancement of anti-tumor activity in most patients. The primary end-point of this trial was designed taking this into consideration and seeks to compare the magnitude of tumor regression of the triplet (navitoclax, dabrafenib, and trametinib) versus the standard of care doublet (dabrafenib and trametinib) in the randomized phase II portion of the study. Further, emphasizing the need to empower trials with tumor tissue analysis to permit predictive biomarker investigations, this study also will compare baseline tumor characteristics and on-treatment effects of treatment to determine potential biomarkers that may predict outcome.

## Supporting Information

Figure S1 **Selected BCL-2 family member mRNA expression in samples from patients before treatment with BRAF inhibition.** Pre treatment expression of *BCL2-L1*, *BCL2-L2*, *BIM*, *BID* and *MCL-1* mRNA from tumors of patients with metastatic melanoma does not correlate with response to BRAF inhibition. (**A–D,F**) Linear regression between duration of patient response to BRAF inhibition and mRNA expression levels relative to endogenous gene *βActin* prior to treatment. (**E**) Excluding an outlier from the linear regression shows a possible trend emerging for *BIM* where higher levels of mRNA expression might be positively associated with duration of treatment (p>0.05).
(EPS)

Figure S2 **The effect of BRAF inhibition, BH3-mimetics or their combination on apoptosis and protein expression levels of BCL2 family members in BRAF^V600E^ melanoma cell lines.** **A**. Fluorescence activated cell-sorting (FACS) for Annexin after indicated drug treatment in BRAF^V600^ cell lines A2058, SKMel28 and Mel526. Drug combinations used at a 1∶1 ratio. **B**. Western blotting of BIM and MCL1 in BRAF^V600^ cell lines A2058, Mel526, UACC903 and UACC257 after 2, 6 and 24 hours treatment with a BRAFi, ABT and the combination of both BRAFi and ABT.
(EPS)

Figure S3 **BIM and Apptosis in Xenografts.** IHC for BIM in xenograft tumors from mice treated with vector, ABT, PLX or the combination of ABT + PLX (**A**) and the detection of apoptosis in these tumors using a TUNEL assay (**B**).
(TIFF)

Table S1 **Patient Characteristics.** Patients with metastatic melanoma containing BRAF^V600E^ mutation (confirmed by genotyping) were enrolled on clinical trials for treatment with a BRAF inhibitor (vemurafenib) or combined BRAF + MEK inhibitor (dabrafenib + trametinib). Listed are patient age, site of disease, treatment, maximum response (RECIST), time to progression (months) and *BCL-2* mRNA levels.
(DOCX)

Table S2 **PCR Primers.** Primers used for RTPCR.
(DOCX)

Methods RPPA S1 **Detailed methods for Reverse Phase Protein Array analysis.**
(DOCX)
