# Supplementary figures and images for: Clinical Profiling of BCL-2 Family Members in the Setting of BRAF Inhibition Offers a Rationale for Targeting De Novo Resistance Using BH3 Mimetics
Source: PLoS One. 2014 Jul 1;9(7):e101286. doi: 10.1371/journal.pone.0101286 (PMC4077767; doi:10.1371/journal.pone.0101286)

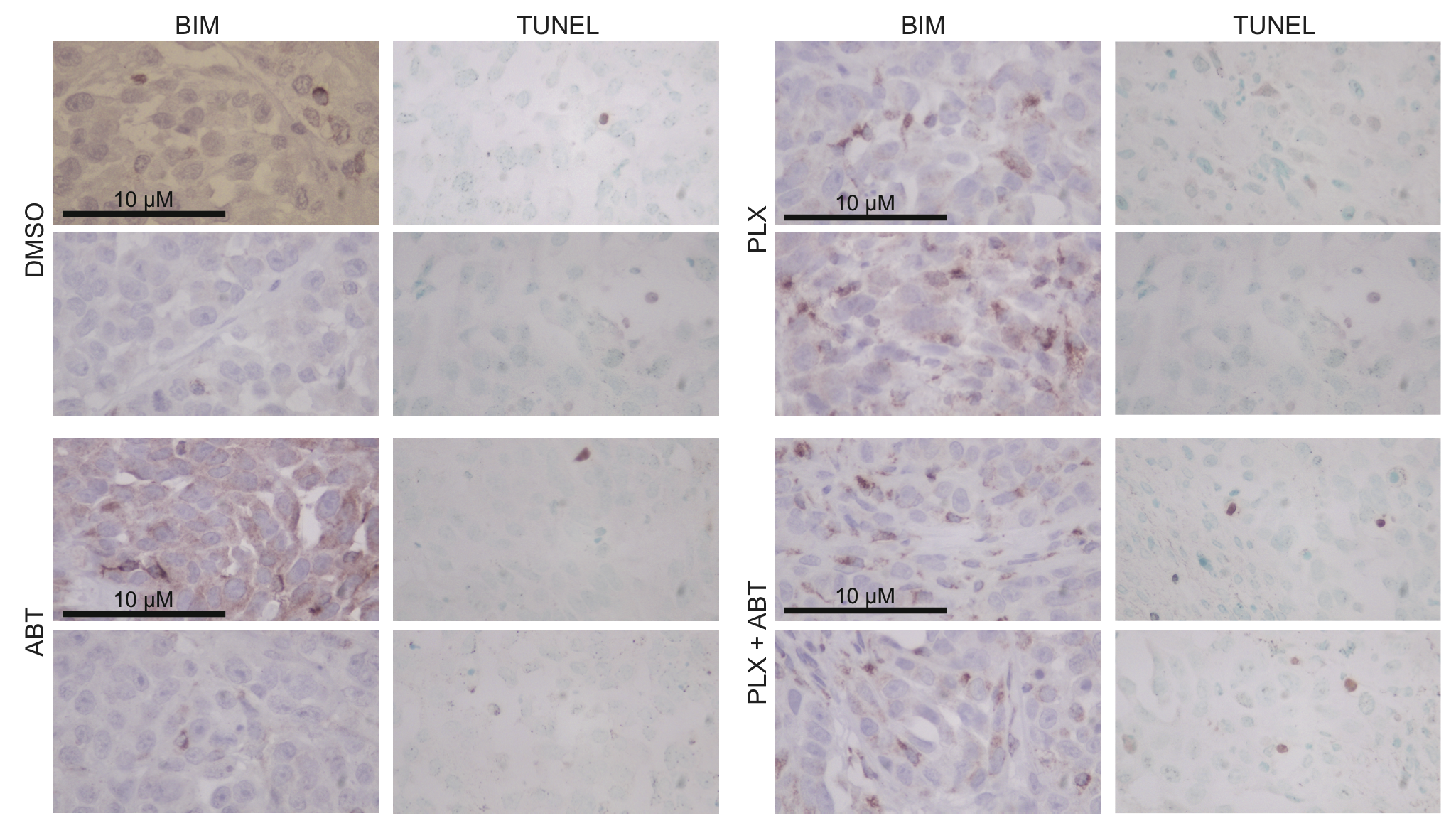

Supplement: Figure S3 — BIM and Apptosis in Xenografts. IHC for BIM in xenograft tumors from mice treated with vector, ABT, PLX or the combination of ABT + PLX (A) and the detection of apoptosis in these tumors using a TUNEL assay (B). (TIFF) [file pone.0101286.s003.tif]
