# Supplementary material for: Clinical Profiling of BCL-2 Family Members in the Setting of BRAF Inhibition Offers a Rationale for Targeting De Novo Resistance Using BH3 Mimetics
Source: PLoS One. 2014 Jul 1;9(7):e101286. doi: 10.1371/journal.pone.0101286 (PMC4077767; doi:10.1371/journal.pone.0101286)
Supplement: Table S1 — Patient Characteristics. Patients with metastatic melanoma containing BRAFV600E mutation (confirmed by genotyping) were enrolled on clinical trials for treatment with a BRAF inhibitor (vemurafenib) or combined BRAF + MEK inhibitor (dabrafenib + trametinib). Listed are patient age, site of disease, treatment, maximum response (RECIST), time to progression (months) and BCL-2 mRNA levels. (DOCX) [file pone.0101286.s004.docx]

| **Patient ID** | **Age** | **Site of disease** | **Treatment** | **Response** | **RECIST** | **Time to Progression (months)** | ***BCL2*  Level Pre-Treatment** | ***BCL2*  Level ON-Treatment** |
| --- | --- | --- | --- | --- | --- | --- | --- | --- |
| 1 | 68 | sc | BRAFi | SD | (-25%) | 5 | NA | NA |
| 2 | 68 | n | BRAFi | PR | (-54%) | 8.5 | 1.12868E-05 | 8.2529E-06 |
| 3 | 42 | sc | BRAFi | SD | (-10%) | 10 | 0.001476776 | 0.000238014 |
| 4 | 49 | lu,sc | BRAFi | PR | (-56%) | 3.5 | NA | NA |
| 5 | 74 | n | BRAFi | SD | (-27%) | 6.5 | 1.17525E-05 | 3.59579E-05 |
| 6 | 73 | sc | BRAFi+MEKi | PR | (-60%) | 21 | 5.62389E-06 | 6.4964E-05 |
| 7 | 56 | sc,n | BRAFi+MEKi | CR | (-100%) | 17 | 0.000353337 | 0.000615196 |
| 8 | 37 | sc,br | BRAFi+MEKi | PR | (-30%) | 3 | 0.000913273 | 0.000499694 |
| 9 | 36 | n,br | BRAFi+MEKi | PR | (-45%) | 7 | NA | NA |
| 10 | 37 | li,sc,n | BRAFi+MEKi | SD | (-13%) | 3 | 0.001433078 | 0.000111037 |
| 11 | 72 | sc,br | BRAFi+MEKi | PR | (-80%) | 10 | 4.49911E-05 | 5.15713E-06 |
| 12 | 31 | sc,br,n | BRAFi+MEKi | PR | (-89%) | 12 | 0.000842326 | 7.97955E-05 |
| 13 | 69 | sc,n | BRAFi+MEKi | PR | (-58%) | 9 | 6.23176E-05 | 0.000257465 |
| 14 | 25 | n,b | BRAFi+MEKi | PR | (-65%) | 8 | NA | NA |
| 16 | 41 | n,sc,lu,li | BRAFi+MEKi | SD | (-19.5%) | 10 | 0.00638986 | 0.00532378 |
| 24 | 61 | br,sc,lu,li,n | BRAFi | PR | (-53%) | 2 | 0.001649983 | 0.000659351 |
| 25 | 50 | n,sc | BRAFi+MEKi | PR | (-64%) | 3 | 2.58406E-05 | 2.89048E-05 |

**Table S1: Patient characteristics**
