# Supplementary material for: Clinical Profiling of BCL-2 Family Members in the Setting of BRAF Inhibition Offers a Rationale for Targeting De Novo Resistance Using BH3 Mimetics
Source: PLoS One. 2014 Jul 1;9(7):e101286. doi: 10.1371/journal.pone.0101286 (PMC4077767; doi:10.1371/journal.pone.0101286)
Supplement: Table S2 — PCR Primers. Primers used for RTPCR. (DOCX) [file pone.0101286.s005.docx]

**Table S2: Primers**

| **Gene** | **Direction** | **Sequence** |
| --- | --- | --- |
| BCL2L1 | Forward | GTC CTC ACT CCC AGT CCA AA |
| BCL2L1 | Reverse | GCT GAG GCC ATA AAC AGC TC |
| BCL2L2 | Forward | TTT GGT TCG GCT TTA TCA GG |
| BCL2L2 | Reverse | GAG GAC TGC GAG TTC CAA AG |
| BIM | Forward | GAT AGT GGT TGA AGG CCT GG |
| BIM | Reverse | CCT CCC TAC AGA CAG AGC CA |
| BID | Forward | GGA ACC GTT GTT GAC CTC AC |
| BID | Reverse | GAG GAG CAC AGT GCG GAT |
| MCL-1 | Forward | CAT TCC TGA TGC CAC CTT CT |
| MCL-1 | Reverse | TCG TAA GGA CAA AAC GGG AC |
| BCL-2 | Forward | CGC CCT GTG GAT GAC TGA GT |
| BCL-2 | Reverse | CCC AGC CTC CGT TAT CCT G |
| ACTB | Forward | GTT GTC GAC GAC GAG CG |
| ACTB | Reverse | GCA CAG AGC CTC GCC TT |
